# Reduced Bioavailability of Antidepressants for the Management of Generalized Anxiety Disorder Following Bariatric Surgery: A Case Study

**DOI:** 10.7759/cureus.56373

**Published:** 2024-03-18

**Authors:** Matthew Schaefer, Fabio Garrote, Patricia Junquera

**Affiliations:** 1 Neurosurgery, Florida International University, Herbert Wertheim College of Medicine, Miami, USA; 2 Anesthesiology, Florida International University, Herbert Wertheim College of Medicine, Miami, USA; 3 Psychiatry and Behavioral Sciences, Florida International University, Herbert Wertheim College of Medicine, Miami, USA

**Keywords:** bioavailability, weight loss, depression, selective serotonin reuptake inhibitors (ssris), gastric sleeve, roux-en-y, bariatric surgery

## Abstract

Bariatric surgery is a commonly performed procedure for patients who have failed to achieve weight loss through medical and lifestyle interventions. However, the altered gastrointestinal anatomy resulting from the surgery can significantly impact the bioavailability of antidepressants in patients with generalized anxiety disorder, potentially leading to uncontrolled anxiety symptoms.

This case report describes a patient with generalized anxiety disorder who underwent Roux-en-Y gastric bypass surgery and subsequently experienced increased anxiety symptoms due to poor antidepressant bioavailability. The patient's medication was adjusted to a sublingual formulation, resulting in improved anxiety control and reduced side effects.

Healthcare providers should be aware of the potential impact of bariatric surgery on medication absorption and closely monitor patients with generalized anxiety disorder for potential psychiatric medication-related complications postoperatively. The use of alternative routes of administration, such as sublingual medication, may be beneficial in improving drug bioavailability and managing anxiety symptoms.

Creating awareness in primary care offices about poor drug absorption and using alternatives such as the sublingual route of administration to achieve optimal systemic delivery requires a multifaceted approach involving education and training for healthcare providers as well as patient education to ensure they are informed and engaged in their own care. By implementing these strategies, primary care providers can improve patient outcomes and prevent unnecessary referrals to specialists.

## Introduction

With obesity being a worldwide public health concern and a primary contributing factor to the leading cause of death in the US, many adults and healthcare providers have partnered through both behavioral and medical interventions to treat this major healthcare epidemic. When medical management and dietary and lifestyle modifications fail, patients often choose to undergo bariatric procedures to meet their health and lifestyle goals. This patient population may choose one of the various types of bariatric procedures, which are typically classified as malabsorptive (Roux-en-Y gastric bypass) or restrictive (gastric sleeve), or a combination of both. Immediately after surgery, the patient may experience rapid weight loss that is often associated with an elated mood because of improved quality of life, improvement or resolution of comorbid conditions, or decreased cognitive dissonance about self-image [1]. Herein we aim to analyze the factors that may affect the postoperative population of patients who continue to take psychiatric medication. 

Roux-en-Y gastric bypass

The Roux-en-Y gastric bypass surgery is a procedure where the distal esophagus is anastomosed directly to the jejunum, bypassing the stomach and the duodenum, thereby decreasing absorption. The first step of the Roux-en-Y gastric bypass surgery is to dissect the distal esophagus from the stomach and create the gastric pouch. Next, the jejunum is divided 40-50 cm distal to the ligament of Treitz at the distal end of the duodenum creating proximal and distal attachment sites. An esophagojejunostomy is created by suturing the distal esophagus to the proximal jejunum’s free end, and a jejunojejunostomy is created by taking two remaining jejunal segments and suturing them together. This maneuver bypasses the stomach and duodenum, eliminating digestion in the stomach and absorption in the duodenum. 

The indications for the Roux-en-Y gastric bypass surgery are twofold. All patients with a BMI above 40kg/m^2^ or patients with a BMI between 35 and 40kg/m^2^ and comorbidities related to obesity such as hypertension, type 2 diabetes, or sleep apnea can undergo gastric sleeve or Roux-en-Y or gastric bypass surgery. The procedure can be performed through open surgery, laparoscopic surgery, or robotic assistance. The number of interventions involving bariatric surgery has steadily increased in the United States over the last five years [2]. Despite this high surgical volume, these procedures have been associated with both short- and long-term complications. Early postoperative complications following Roux-n-Y gastric bypass involve hernias, fistula formation, dumping syndrome, small bowel obstruction, hypoglycemia, ulcer formation, and gastric remnant distension [3].

Psychological evaluation is needed before a patient can be cleared for this surgery. The assessment is performed through a clinical interview and psychological testing. According to Snyder, no standardized psychological evaluation exists but clinical interviews paired with psychological testing are generally used. The interview consists of reasons for seeking the surgery, weight, height, and BMI history, current eating behaviors, understanding the surgery and lifestyle changes, social support and social history, and psychiatric history. The most widely used psychological tests for objective outcomes are the Minnesota Multiphasic Personality Inventory-2 and Millon Behavioral Medicine Diagnostic. The interview and objective assessments are then analyzed and discussed with the surgeon who will be performing the case [4]. 

Sleeve gastrectomy

A sleeve gastrectomy or gastric sleeve is a bariatric procedure that results in the removal of the greater curvature of the stomach and fundus, with the post-operative anatomical remains being the lesser curvature and the antrum [5]. Most commonly, the laparoscopic form of this procedure requires 5 to 6 trocars, and a 32-48 French bougie via the esophagus. The partial gastrectomy is performed with staples, starting 6 cm from the pylorus and moving in a cephalic version toward the fundus until reaching the angle of His [5]. 

Indications for surgical intervention involving sleeve gastrectomy as like those of Roux-n-Y and are reflected in the 1991 National Institutes of Health (NIH) consensus conference criteria. Gastric sleeve is indicated for those individuals with BMI ≥40 kg/m^2^, or BMI between 35 and 40 kg/m^2^, and at least one weight-related comorbidity [6]. Moreover, clinical practice guidelines as of 2019 mention that gastric sleeve is only indicated for BMI between 30 and 35 kg/m^2^ when patients had either uncontrolled diabetes or metabolic syndrome [7]. Sleeve gastrectomy has the advantage of a low complication profile but the literature has described bleeding, stenosis of the stroma, and gastric leaks as the most common unwanted post-operative outcomes [8]. Moreover, multiple cases have reported intractable severe gastric acid reflux as a late complication of sleeve gastrectomy [9]. As previously reported by Snyder, despite the lack of a standardized tool, psychological evaluation for procedure clearance is required for all patients prior to bariatric surgery [4]. The same tools used for psychological evaluation in Roux-n-Y gastric bypass can be applied to sleeve gastrectomy. 

Drug bioavailability

The surface of the gastrointestinal tract, specifically the intestinal epithelium, plays a crucial role in the absorption of nutrients and drugs from the digestive tract into the bloodstream. In response to various stimuli, of internal or external nature, the intestinal epithelium can undergo physiological and morphological changes. This specialized epithelial layer can undergo changes in the expression of transporters and enzymes that determine the absorption of specific nutrients and drugs [10]. The effects of bariatric surgery on drug bioavailability can vary depending on several factors, including the type of surgery performed, the properties of the drug, and the drug product formulation, resulting in some drugs being more susceptible to physiological decline following surgery [10]. 

Given the nature of the operations performed above, there are various factors that may affect a patient’s ability to absorb different medications. These factors include the type of surgery, which may affect the time of transit through the alimentary canal. Patients with a disrupted or an obliterated pylorus as seen in patients with gastric bypass surgery do not have autonomic regulation and they also lack the physiologic intestinal milieu that is required for proper absorption to take place. Moreover, those patients undergoing bariatric surgery have a significant reduction in the surface area of the portion of the gastrointestinal tract involved with securing absorption of food and medications. In this paper, we will be discussing a case that resulted in reduced efficacy of antidepressant therapy for the management of generalized anxiety disorder following Roux-n-Y gastric bypass and sleeve gastrectomy. 

Anxiety is a common condition affecting compromised patients in primary practices as well as hospital settings. To provide effective treatment and optimal quality of care, psychiatrists and primary care physicians should be aware of the alternatives for delivering psychiatric drugs. The diagnosis of generalized anxiety disorder requires patients to experience excessive worrying that is not focused on a single specific fear, and it must last at least six months [11]. First-line treatment for adults involves cognitive-behavioral therapy, involving exposure procedures to ameliorate symptomatology. In terms of pharmacotherapy, FDA-approved medications are venlafaxine XR, duloxetine, paroxetine, and escitalopram [12]. Serotonin reuptake inhibitors (SSRIs) are easily absorbed in the gastrointestinal tract, primarily the small and large intestines, and then go through first-pass metabolism [13]. Following first-pass metabolism their maximal concentration in serum is achieved within a maximum of eight hours, varying among different patients [14]. Since SSRIs are highly lipophilic and dependent on stomach's acidic environment to be properly absorbed, a lower blood level concentration is achieved now. A factor interfering with SSRI absorption in the small and large intestines is the reduction of gastric acid production following gastric bypass surgery, which decreases their solubility and thus their bioavailability [15].

A safe, logical alternative for these patients would be to deliver medications through a different route. Rapid absorption, improving the bioavailability of antidepressants can be achieved via sublingual delivery in patients unable to fully benefit from oral administration [16]. The effectiveness of this delivery route is associated with the permeability of the mucosa under the tongue, which provides a large network of capillaries, allowing for rapid absorption and bypassing the first-pass metabolism of the drug being delivered [16]. The literature provided examples of patients with issues related to gastrointestinal malabsorption, and diagnosis of major depressive disorder, receiving effective treatment with sublingual SSRIs. Two case reports were of increased significance since the plasma drug level was found within the therapeutic range while using a sublingual delivery system. In these two cases, both patients received effective treatment resulting in the resolution of their symptoms and discharge [17]. Additionally, rectally administered pharmacotherapy might be also taken into consideration for patients unable to absorb medication through intestinal absorption. Although this is a potentially viable option, many patients prefer to avoid this route of administration, and the literature on rectal preparation for antidepressants is limited [16].

## Case presentation

The patient was a 40-year-old female who experienced significant anxiety symptoms, which had not improved with their current medication by mouth (PO) Escitalopram (Lexapro). She reported stressors related to family and work made her anxiety worse. Specifically, the patient stated she felt bullied by her boss which exacerbated her anxiety. The patient has a history of microcytic anemia, panic attacks, and generalized anxiety disorder. The patient denied symptoms of mania/hypomania, psychosis, and persecutory delusions. The patient did not have visual or auditory hallucinations. She denies suicidal or homicidal ideation. Surgical history is significant for Roux-en-Y gastric bypass five months prior to the psychiatric appointment. The poor response to current medication may be related to poor absorption due to gastric bypass surgery. She was started on soluble sublingual benzodiazepine PRN for panic attacks and escitalopram (Lexapro) 10mg in liquid solution q24hrs for anxiety management. Vitamin B12 injections and iron liquid solution are also being considered for the patient's anemia. The patient is currently on 20 mg of Escitalopram (Lexapro) oral solution and has been stable. The patient has only needed clonazepam (Klonopin) 0.25 dissolvable tablet sublingual when traveling for work. Exploring ways to manage or reduce the impact of stressors on the patient's anxiety symptoms may be helpful. Additionally, monitoring the patient for potential side effects of the medications and adjusting the treatment plan as needed is important for optimal management of her anxiety. For this patient, we see that switching her SSRI route from PO to sublingual and dissolvable tablets made a positive impact.

## Discussion

Receiving medications orally post gastric bypass has hindered the patient from absorbing a therapeutic dose of their SSRI. The National Institute of Health discusses specific patient populations for Escitalopram, such as patients with hepatic impairment and patients with renal impairment, but it does not discuss patients with gastric bypass [18]. This patient group is at risk of absorbing a subtherapeutic dose because of the gastric bypass surgery. Alalwan et al. in their study discussed how certain medications were impacted by bariatric surgery, with oral escitalopram among them, noting that Roux-en-Y gastric bypass decreased the bioavailability [19]. Hamad et al. stated that 8 out of the 12 patients in their study taking SSRIs had reduced drug bioavailability one month after Roux-en-Y gastric bypass but that drug bioavailability started to normalize around 6 months after surgery [15]. By six months, 10 of the 12 patients had detectible blood concentrations of their SSRI medication. This can be compared to our patient who experienced psychiatric symptoms five months post bariatric surgery. She was switched to 20 mg of escitalopram (Lexapro) oral solution and has been stable. Furthermore, Marzinke et al. stated in their research that four study participants had decreased concentrations of escitalopram with drug serum concentrations decreasing a mean of 33% (4%-71%) at two weeks postsurgery and more at six weeks [20]. This study did not follow the patients at six months postsurgery which would have been an important time frame to analyze, based on previous literature. Understanding drug absorption post gastric bypass is an important concept, but it is also critical to understand the recommendations for patients taking medications after the procedure. Lorico’s paper discussed medical management recommendations for patients [21]. A recommendation in the literature is that the patient in this report can likely benefit from using nonoral medications, but instead switching to a sublingual form. 

## Conclusions

This case report shows that there are challenges with medication absorption in postbariatric surgery patients, notably SSRIs. Understanding the complex effects of these procedures on drug bioavailability is crucial. Exploring alternative medication delivery methods is essential for optimizing patient care. Vigilant monitoring by healthcare providers is necessary to detect complications like antidepressant discontinuation syndrome. Adjusting treatment plans is vital for achieving the best possible mental health outcomes in post-bariatric surgery patients. The future indications should be for primary care physicians and psychiatrists to thoroughly discuss a patient's surgical history when taking antipsychotics so they can adjust the dose and or method of delivery for these medications. Lastly, bariatric surgeons should review a patient's medication list and psychiatric history so they can counsel the patient regarding their medical management postbariatric surgery.

## References

[1] Vaishnav M, Gupta S, Vaishnav P (2022). Psychiatric intervention pre- and post-bariatric surgery. Indian J Psychiatry.

[2] (2024). Estimate of Bariatric Surgery Numbers, 2011-2022. https://asmbs.org/resources/estimate-of-bariatric-surgery-numbers/.

[3] Jammah AA (2015). Endocrine and metabolic complications after bariatric surgery. Saudi J Gastroenterol.

[4] Snyder AG (2009). Psychological assessment of the patient undergoing bariatric surgery. Ochsner J.

[5] Katz DP, Lee SR, Nachiappan AC (2011). Laparoscopic sleeve gastrectomy: a guide to postoperative anatomy and complications. Abdom Imaging.

[6] Seeras K, Sankararaman S, Lopez PP (2024). Sleeve gastrectomy. StatPearls [Internet].

[7] Mechanick JI, Apovian C, Brethauer S (2019). Clinical practice guidelines for the perioperative nutrition, metabolic, and nonsurgical support of patients undergoing bariatric procedures - 2019 update: cosponsored by American Association of Clinical Endocrinologists/American College of Endocrinology, The Obesity Society, American Society For Metabolic & Bariatric Surgery, Obesity Medicine Association, and American Society of Anesthesiologists. Endocr Pract.

[8] Gagner M, Deitel M, Kalberer TL, Erickson AL, Crosby RD (2009). The Second International Consensus Summit for sleeve gastrectomy, March 19-21, 2009. Surg Obes Relat Dis.

[9] Langer FB, Bohdjalian A, Shakeri-Leidenmühler S, Schoppmann SF, Zacherl J, Prager G (2010). Conversion from sleeve gastrectomy to Roux-en-Y gastric bypass--indications and outcome. Obes Surg.

[10] Steenackers N, Vanuytsel T, Augustijns P (2021). Adaptations in gastrointestinal physiology after sleeve gastrectomy and Roux-en-Y gastric bypass. Lancet Gastroenterol Hepatol.

[11] (2016). Impact of the DSM-IV to DSM-5 Changes on the National Survey on Drug Use and Health [Internet]. National Library of Medicine- Substance Abuse and Mental Health Services Administration Center for Behavioral Health Statistics and Quality.

[12] Chu A, Wadhwa R (2024). Selective serotonin reuptake inhibitors. StatPearls [Internet].

[13] (2024). Selective serotonin reuptake inhibitors: Pharmacology, administration, and side effects. https://medilib.ir/uptodate/show/14675.

[14] Kam PC, Chang GW (1997). Selective serotonin reuptake inhibitors. Pharmacology and clinical implications in anaesthesia and critical care medicine. Anaesthesia.

[15] Hamad GG, Helsel JC, Perel JM (2012). The effect of gastric bypass on the pharmacokinetics of serotonin reuptake inhibitors. Am J Psychiatry.

[16] Kaminsky BM, Bostwick JR, Guthrie SK (2015). Alternate routes of administration of antidepressant and antipsychotic medications. Ann Pharmacother.

[17] Pakyurek M, Pasol E, Brook S (1999). Sublingually administered fluoxetine for major depression in medically compromised patients. Am J Psychiatry.

[18] Landy K, Rosani A, Estevez R (2024). Escitalopram. StatPearls [Internet].

[19] Alalwan AA, Friedman J, Alfayez O, Hartzema A (2022). Drug absorption in bariatric surgery patients: a narrative review. Health Sci Rep.

[20] Marzinke MA, Petrides AK, Steele K (2015). Decreased escitalopram concentrations post-Roux-en-Y gastric bypass surgery. Ther Drug Monit.

[21] Lorico S, Colton B (2020). Medication management and pharmacokinetic changes after bariatric surgery. Can Fam Physician.

